# Progress in Medicine

**Published:** 1897-05-29

**Authors:** 


### Progress in Medicine.

### FEVERS.

**Typhoid.**—Among the many improvements of the serum test Professor Wright's substitution of dead for living cultures is one of immense practical value.[1] When living cultures are used the test is inapplicable unless there is a laboratory at hand and growing cultures ready, but the dead cultures can be kept sealed up in capsules or sent by post. He finds, in fact, that emulsions of fresh agar cultures killed by exposure to a temperature of 60 deg. C. for five or ten minutes, sealed, and kept for some weeks, react with typhoid serum just as well as living ones, becoming "clumped," or in Wright's tubes giving a floccular sedimentation quite distinct from the fine granular deposit of dead bacteria which occurs without the addition of serum. The latter test can be observed without a microscope, so that the whole apparatus is reduced to a supply of capsules of dead cultures, glass tubing, and a blowpipe for making it into capillary tubes. For the blowpipe an ether spray filled with methylated spirit and lighted will suffice. The details as to the tubes are given in the *British Medical Journal* of January 16th, and the method is applicable to both typhoid and Malta fever microbes. Jemma,[2] in using the large quantity of serum which Widal first proposed, obtained the reaction without fail in twelve typhoid cases, but especially definitely at the acme of the fever, and remarks that the properties of the typhoid serum are contained in or due to the fibrinogen and globulin alone, and are destroyed by heating to 70 deg. C. for ten minutes. Herman M. Biggs and Park[3] mention that exposure to sunlight for several weeks does not destroy the power of the serum. Up to January 1st 422 cases of supposed typhoid had been tested by various observers, and 95 per cent. responded to the serum test. Biggs and Park then found that with serum of normal individuals, if more than one part of serum to nine of the culture was used, reactions frequently occurred, but not otherwise in 308 picked cases of non-typhoid diseases. Their Health Department at New York followed the example of Wyatt, of Montreal, in providing means for the local physicians to have the test applied to any suspected case at the public laboratories. Two drops of blood are placed on a slide sent round by the Health Department and allowed to dry. The slides are collected, the blood moistened with about five times as much water, a drop of culture added, and the mixture examined under a one-sixth objective for clumping. They also notice that in dried blood of healthy persons, if too concentrated, there may be immobilisation, and if there is much dust the bacilli may be entangled in it and a false appearance of clumping produced. Thus the best results are to be obtained from the serum of a blister, if the slight delay in obtaining it is of no importance. In all cases, however, if an accurate result is to be obtained, not more than one part of serum to nine of the broth culture must be used. According to the New York procedure the test is applied with a one in ten dilution, and if successful then with greater dilutions. When absent with a one in two delution the proof against typhoid is very high. If there is no change in motility of the bacilli within five minutes in a one in ten dilution, even if slight clumping occur later, it is not regarded as a typhoid reaction. As to results, the Department record the examination of 140 cases believed to be typhoid, in 100 of which they obtained a positive reaction with a one in ten dilution, but they note one definite case from the spleen of which typhoid bacilli were obtained which failed in this test for at least three weeks. On the other hand, of a hundred healthy persons not one responded to the test. The results in persons suffering from other diseases showed very few who gave a marked immediate reaction, and in these the diagnosis was doubtful. Still there is a reaction, though a delayed one, in some diseases, and they regard typhoid as only possessing the same reaction in a very high degree. The quantitive limits of this power in typhoid are little known, but they estimate that over two-thirds of all typhoid cases reach the standard they have fixed by the end of the first week, while only two per cent. of non-typhoid ones do so, and few if any of these latter react in dilutions of one to forty. They insist on the importance of the exact method and time being stated in reports of the use of this test.

Haedke[4] compares the various tests for typhoid. Puncture of the spleen and examination of the blood is fairly certain, but undesirable in practice. Elsner's test does not always discover the bacilli in the stools. Widal's reaction has given him good results, especially the bouillon test as opposed to the microscopic one. He adds fixed proportions of serum and culture to a broth tube and finds a flocculent precipitate in six to eight hours. In 22 cases of typhoid he obtained a positive result from all. He uses as a preliminary test the immediate stoppage of movement which is seen when bacilli have typhoid serum added to them.

J. T. Mackenzie[5] reports, in the *Canadian Practitioner*, 82 cases tested by the dried blood method, and obtained a positive result in 57 out of 61 cases of typhoid, and a negative one in all other diseases. He uses such a dilution that not more than 50 to 100 bacilli appear on the field, taking care to have a very fresh culture. He apparently allows reactions which occur even after several hours.

H. E. Durham[6] notices two points, first that a positive result cannot always be obtained, and secondly, that it is given by Gärtner's bacillus of enteritis as well as by typhoid. Grünbaum too insists on the importance of accurate dilution of the serum and the observance of a time limit, but he had not come across any case of typhoid in which the reaction was not present at some period of the disease. He had even produced clumping of the non-motile diplococci in scarlatina by the use of serum from another patient suffering from the same complaint, while Durham distinguishes between anti-bacterial and anti-toxic serums both of which may possess some "clumping" power. N. E. Roberts asks whether there is not some degree of immunity produced in the early days in which this clumping is shown in typhoid. Though it does not prevent relapses, patients do go through relapses far better than we should have expected. He therefore holds that, though no thoroughly protective immunity is developed in early days, gradual immunisation is being built up ; that is, an anti-toxic power is gradually added to the anti-bacterial.

Wyatt Johnston and Mctaggart have supplemented their previous papers by observations on 500 cases.[7] They distinguish between clumping, paralytic and disintegrative powers, and find that the simplicity of the dried blood method as originally described does not require any modification for accurate work, provided that weak, non-virulent cultures are used. Under this condition they can dispense with the necessity for accurate dilution insisted on by so many authors who use virulent cultures. They obtain for their tests a drop of twenty-four hour bouillon from a weak stock grown at the temperature of the room, and transplanted once a month. For all practical purposes a drop of water from a dried blood clot added to this bouillon will give correct reaction, provided that the mixture is freely fluid and not syrupy. Pick, of Prague,[8] also writes in praise of the dried blood method, and finds that when the serum of normal individuals is added to cultures there may sometimes be agglutination but never the complete reaction.

The experiments of Pfeiffer and Kolle[6] show that substances are present in the serum of typhoid convalescents which are actively bactericidal, though not anti-toxic, for the typhoid bacillus alone; and similar substances exist in the serum of goats which have been treated by increasing injections of those bacilli. They discovered further that by injections of dead bacilli human beings could be rendered immune to typhoid, and even after a single injection their serum causes clumping of bacilli just the same as that of a patient suffering from typhoid[9]. Wright and Semple[10] have elaborated a method of anti-typhoid vaccination. They kill an emulsion of typhoid bacilli in sterilised capsules by exposure to a heat of 60 degrees for five minutes, and inject into the flank of a human being from a twentieth to a quarter of a capsule, the latter amount being fatal to a ten-ounce guinea-pig. Some tenderness and congestion appears about the site of injection, and lasts two days. Vomiting, fainting, and pyrexia last for one or more days. After this had been carried out on 18 persons it was found that the blood of these persons caused "clumping" of typhoid bacilli; but even when this power is strongly developed it does not follow that the blood will actually kill any bacilli which enter the system. Nevertheless, there is some proof that such patients are immune to the disease for the time. The fact that "clumping" is shown in all typhoid patients in the early stages probably only means that a gradual immunity is being worked out which may be too late after the infection to save them. One patient after vaccination showed no bad symptoms when inoculated with living typhoid microbes, but proof as regards the others must await accidental verification. The writers believe that the immunity may last some years, and its existence can be tested by the clumping test.

Four cases of existing typhoid were treated with antitoxin prepared by Mr. T. J. Bokenham, and are reported by F. M. Pope,[11] who considers that the serum had undoubtedly a beneficial result. Defervescence commenced sooner than in other patients, and the general state of the patients was improved. From 5 to 10 cub. c. were injected on three or more occasions. Another case was treated in the same manner by P. R. Cooper.[12] Intestinal hæmorrhage, hæmoptysis, hiccough, and a marked "typhoid state" showed the extreme gravity of the case. Injections of 5 cub. c. of serum were followed by definite improvement at once and fall of temperature, which became permanent after a third dose of 8 cub. c. Thus it seems clear that some degree of assistance in the struggle with the invading microbes is given by the administration of this serum, and future results will be awaited with much interest. We may here refer to some observations on the Diazo test for typhoid. Jey[13] has recorded three thousand experiments to test its value, and finds it occurs with fair regularity in typhoid from the second to the seventh day, more or less markedly as the severity of the disease varies; and it does not appear in malaria or inflammatory states of the stomach and bowels. In tuberculosis heart and kidney disease it betokens a grave issue, and he believes that the reaction in typhoid is due to some products of the typho-toxin.

The diagnosis of Malta fever from typhoid, malaria, and other forms of continued fever is shown to be easily made by the Widal serum test. For this A. E. Wright and F. Smith have employed the tubes devised originally for typhoid. They found[14] by this means evidence of Malta fever contracted in Northern India, and probably also in Hong Kong. Incidentally, too, they noted the "clumping" power retained after typhoid for twelve, and after Malta fever for three years.

Horton Smith[15] has never been able to find typhoid bacilli in the urine during the first week, but has confirmed the appearance of them later by Widal's test and other methods to differentiate them from the colon bacillus. Hence the importance of disinfection of the urine and chamber utensils during typhoid. Remlinger and Schneider appear, however, to show the presence of a bacillus agreeing in all, or almost all, respects with Eberth's, in the soil and water, and in the fæces of non-typhoid patients, and, according to McWeeney,[16] they controlled their results by the serum test and by injections into guinea-pigs. If their view is true the organism is most widely diffused, and only needs favourable circumstances or predisposition of patients to cause an outbreak.

[1] B. Med. Jour., May 15. [2] B. Med. Jour., March 10. [3] Amer. Jour. Med. Sciences, March. [4] B. Med. Jour., Feb. 20. [5] Lancet, Feb. 6. [6] Lancet, Feb. 13. [7] N. Y. Med. Jour., Feb. 27. [8] Med. Chron., Feb. [9] Amer. J. Med. Sciences, March. [10] B. Med. Jour., Jan. 30. [11] Ibidem. [12] B. M. J., Feb. 27. [13] Wiener Med. Woch., Dec. 19. [14] Lancet, March 6. [15] Lancet, Feb. 13. [16] B. Med. Jour., March 13.

